# Editorial: Prognostic factors in non-small cell lung cancer

**DOI:** 10.3389/fonc.2023.1174625

**Published:** 2023-03-14

**Authors:** Mohamed Rahouma, Massimo Baudo, Jun Zhang, Luciano Mutti

**Affiliations:** ^1^ Department of Cardiothoracic Surgery, Weill Cornell Medicine, New York, NY, United States; ^2^ Surgical Oncology Department, National Cancer Institute, Cairo University, Cairo, Egypt; ^3^ Department of Cardiac Surgery, Spedali Civili di Brescia, Brescia, Italy; ^4^ Division of Medical Oncology, Department of Internal Medicine, University of Kansas Medical Center, Kansas City, KS, United States; ^5^ Department of Cancer Biology, University of Kansas Medical Center, Kansas City, KS, United States; ^6^ Sbarro Institute for Cancer Research and Molecular Medicine, Center for Biotechnology, College of Science and Technology, Temple University, Philadelphia, PA, United States; ^7^ Department of Applied Clinical Sciences and Biotechnology, L’Aquila University, L’Aquila, Italy

**Keywords:** immune checkpoint inhibitors, prognosis, survival, immunotherapy, lung cancer, noncoding RNA

Lung cancer is the most frequent cause of cancer deaths, and non-small-cell lung cancer (NSCLC) accounts for 85% of all lung cancer cases (1). In 2023, 1,958,310 new cancer cases and 609,820 cancer deaths are projected to occur in the United States, including approximately 350 deaths per day from lung cancer (2). It is the second most frequently diagnosed cancer in both males and females, while the first for number of deaths in both sexes (2). Besides and in comparison to the most common cancers, malignancy-associated suicide risk is the highest among patients with lung cancer, particularly elderly, widowed, male patients and patients with unfavorable tumor characteristics (3). Currently, different cancer treatment options are available such as surgery, chemotherapy, radiotherapy, targeted therapy, immunotherapy, or combination of these. However, there are many patients who receive therapy from which the benefit is minimal or even absent, whilst they do experience treatment-related toxicity. This has raised the need to improve the outcomes by selecting the best patient for an anticancer treatment through prognostic and predictive biomarkers investigation especially in the current personalized medicine era. Prognostic factors provide information about the patients’ overall cancer outcome, regardless of therapy. On the other hand, a predictive biomarker gives information about the effect of a therapeutic intervention, i.e. the treatment benefit (4).

This Research Topic in Frontiers in Oncology “*Prognostic Factors in Non-Small Cell Lung Cancer*” comes to shed light on potential treatment target for NSCLC through the identification of the

- prognostic value of tumor pathological characteristics,
- prognostic gene signatures,
- non-coding RNAs (ncRNA) as prognostic biomarkers, and
- immune-related prognostic biomarkers.

A total of 12 studies analyzed non-coding RNA as possible prognostic biomarkers (Chen et al., Chen et al., Duan et al., Jiang et al., Jiang et al., Li et al., Li et al., Li et al., Tang et al., Wang et al., Weng et al., Yuan et al.). Long non-coding RNAs (lncRNAs) are a particular type of RNA transcripts that don’t code for proteins but are involved in the regulation of critical biological processes and cellular behavior (5). Besides, lncRNAs influence gene expression and have essential roles in carcinogenesis (6). MicroRNA (miRNA) refers to a class of short endogenously ncRNAs that negatively regulate mRNA expression by binding the complementary sequences in the target genes’ 3′-untranslated region (7). Similar to lncRNA, miRNA was shown to be of paramount importance in cancer development and progression. Both these forms of ncRNA interact and compete with each other as competing endogenous RNA (ceRNA) to indirectly regulate downstream target mRNA expression (8).

Our current Research Topic identified various lncRNAs (Chen et al., Jiang et al., Li et al., Li et al., Weng et al.) and miRNA (Duan et al., Jiang et al.), whose up- or down-regulation in cancer tissue compared to normal tissue were associated with adverse clinical outcomes and prognostic models were built upon. Interestingly, five papers (Chen et al., Li et al., Tang et al., Wang et al., Yuan et al.) were able to analyze the ceRNA network (lncRNA-miRNA-mRNA) demonstrating high performance in predicting the survival and chemotherapeutic responses of low- and high-risk patients.

The focus of other 12 papers were the genes and related coded proteins. Elevated expression levels of phosphoenolpyruvate carboxykinase 1 (Shao et al), ASPM, CCNB2, CDCA5, PRC1, KIAA0101, and UBE2T (Chen et al.), soluble Programmed Death Ligand 1 (PD-L1) (Liao et al.), HMMR (Jiang et al.), four particular genes in the 7-methylguanosine-related gene signatures (Lu et al), eighteen endoplasmic reticulum stress-related genes (Shu et al.), two M2 macrophage-related genes (GRIA1 and CLEC3B) (Xu et al.), and Extra Spindle Pole Bodies-Like 1 (Nie et al.) were associated with poorer overall survival. On the other hand, lower expression levels of TTN (Chen et al.) was also associated with worse survival. Of note, Prelaj et al. published the first case of a patient affected by metastatic NSCLC harboring an EGFR exon 20 insertion mutation who achieved a complete response under treatment with poziotinib. This affirms that certain mutations such as EGFR exon20ins has predictive value in certain targeted therapy.

Two cell death paths were further investigated: ferroptosis and cuproptosis. Ferroptosis is a type of oxidative cell death presented in neurological disorders, blood diseases, and tumors that has been proposed as a promising target for killing cancer cells that are resistant to conventional treatment. In their paper, Wang et al. constructed a predictive model of ferroptosis related gene showing that the low-risk group in their model might profit more from immune checkpoint inhibitors. On the other hand, cuproptosis is a novel programmed cell death pathway different from apoptosis and characterized by mitochondrial copper-dependent functional destruction. Wu et al. were able to construct a nomogram for predicting patients’ prognosis from cuproptosis-associated clusters.

As far as pathological features, new insights on patients’ subdivision were analyzed. Morphological changes in the bronchial epithelium (e.g. basal cell hyperplasia, squamous cell metaplasia, and dysplasia) away from tumor foci were associated with a high-risk of distant metastasis and less 5-year metastasis-free survival (Pankova et al.). Besides, tumor-related atelectasis was associated with shorter overall survival (Wang et al.), while adjuvant chemotherapy seems to be beneficial in the survival of stage I lung adenocarcinoma patients with tumor spread through air spaces after resection (Xie et al.). Patients with rib and parietal pleura invasion were also assessed. Patients with rib invasion had poorer survival than those with the invasion of parietal pleura. In addition, in the cohort for parietal pleura invasion, patients were further classified by tumor size and patients with tumor size >5cm had less satisfactory survival outcomes than those with tumor size ≤5cm (Wu et al.). The development of a nomogram of patients with stage I to IIIA NSCLC using machine learning based on radiomic features extracted from computed tomography images and clinicopathological factors suggested that stage IIB -IIIA patients didn’t show any statistically significant difference in the survival rate, irrespective of administration of adjuvant chemotherapy, while stage I-IIA patients displayed a poorer prognosis in patients who had received chemotherapy (Yang et al.). This is arguably not in line with most of the guidelines, and might be related to the technique of analysis used by the authors therefore remains debatable. Of note, Meng et al. analyzed and developed a nomogram model in patients for lung adenocarcinoma (LUAD) with 1 to 5 bone-only metastases, reporting that adding alkaline phosphatase, albumin and leukocyte would improve the predictive accuracy of survival. The prognosis of non-small cell lung cancer patients with central nervous system (CNS) metastasis is poor and the study by Gao et al. deepened the knowledge of this subset of lung cancer patients. They observed that patients with ≥5 CNS metastases or that were developed during treatment were independent risk factors for poor survival. However, radiotherapy for CNS metastasis showed a survival benefit in the entire group, and in patients with driver mutations that can be treated by available targeted therapy such as EGFR, ALK, ROS-1, RET, and other mutations. Despite occurring mostly in the salivary glands, mucoepidermoid carcinoma may occur also in the lungs. When analyzing pulmonary mucoepidermoid carcinoma, stage IV, degree of differentiation (high grade), and lymph node metastasis were associated with worse survival (Li et al.).

Finally, some papers in this Research Topic evaluated different therapeutic strategies or analyzed their outcomes. Luo et al. investigated the adjuvant use of Chinese herbal medicines preparations in the treatment of primary NSCLC. Although Chinese herbal medicines are quite often used in China, such treatments are unlikely to be generalized in other countries, therefore data interpretation has to be careful. PD-1/PD-L1 immune-checkpoint blockade is becoming widely used for metastatic NSCLC. Nevertheless, the pathway inhibited by these drugs is a biological mechanism that prevents autoimmunity when prolonged and/or repeated exposure to the same antigens occurs (9, 10). Fameli et al. reported in their single-cell cytometry study that there is an increase in antinuclear antibodies (ANAs) and extractable nuclear antigen (ENA) antibodies, and that the increased immune-related adverse events were associated with the deregulation of specific immune subpopulations. Ma et al. constructed a nomogram based on patients with metastatic LUAD undergoing primary surgery and three different risk groups could be identified based on the risk score that was constructed using age, gender, primary location, N stage, bone metastasis, liver metastasis, radiotherapy, and chemotherapy.

The brief overview of the articles included in this Research Topic provides important updates regarding prognostic factors in NSCLC from new ncRNAs and their interaction with the related genes, possible subclassification of patients by pathological features, and new insights of current therapies. Eventually it results in a particularly intriguing question regarding how to apply novel techniques, e.g. machine learning and radiomics possesses to improve the current clinical practice.

## Author contributions

All Authors (MR, MB, JZ, LM) participated in writing, editing and reviewing the final manuscript.
